# Reconocimiento de Saberes Tradicionales en Salud. Andes, Antioquia 2019: Aproximación Cualitativa

**DOI:** 10.15649/cuidarte.1307

**Published:** 2021-09-21

**Authors:** Gonzalo Hernando Jaramillo-Delgado, Mariana Agudelo-Arias, Juliana Jaramillo-Vargas, Francia Elena Moreno-Villa

**Affiliations:** 1 Universidad de Antioquia, Medellín, Colombia. Email:hernando.jaramillo@udea.edu.co Universidad de Antioquia Colombia hernando.jaramillo@udea.edu.co; 2 Universidad de Antioquia. Medellín, Colombia. Email: mariana.agudeloa@udea.edu.co Autor de correspondencia Universidad de Antioquia Colombia mariana.agudeloa@udea.edu.co; 3 Universidad de Antioquia. Medellín, Colombia. Email: juliana.jaramillov@udea.edu.co Medellín Colombia juliana.jaramillov@udea.edu.co; 4 Universidad de Antioquia. Medellín, Colombia. Email: francia.moreno@udea.edu.co Medellín Colombia francia.moreno@udea.edu.co

**Keywords:** Medicina tradicional, Etnobotánica, Terapias Espirituales, Etnografía., Medicine, Traditional, Ethnobotany, Spiritual Therapies, Ethnography., Medicina Tradicional, Etnobotânica, Terapias Espirituais, Etnografia.

## Abstract

**Introducción:**

revalorizar y resignificar la identidad de los saberes tradicionales en salud es retador, en una cultura mediada por la industrialización, perviviendo con un pasado histórico, patrimonial cultural y simbólico, desde una identidad mixturada que se conjuga en un sincretismo territorial, que persiste a través de huellas físicas y culturales de tradiciones mágico religiosas.

**Objetivo:**

reconocer las particularidades etnográficas de los agentes portadores de saberes populares en salud que les permite afianzar sus prácticas en el municipio de Andes- Antioquia. Y resignificar estas prácticas para recuperar sus entornos identitarios y rituales simbólicos, en la configuración de su identidad como cuerpo orgánico en los espacios sociales.

**Metodología:**

investigación cualitativa, de tipo descriptivo, con perspectiva etnográfica (microetnográfica), sistematizada a través de entrevistas a profundidad acompañadas de registros fotográficos y observaciones participantes.

**Resultados:**

se reconocen 48 agentes sociales entre 30 y 70 años, la mayoría asentados en la ruralidad. Entre ellos: sobanderos con secreto, sobanderos con dolor o componedores, hierbateros y rezanderos. Se reconoce su identidad diversa desde una emergencia mágica religiosa y se exponen las características de sus saberes heredados, en una ritualidad envolvente que los recrea y vitaliza en los espacios sociales.

**Discusión:**

se analizan los resultados en el marco del espacio social, multidimensional y complejo, a través de sus tres elementos: estructuras sociales, relaciones sociales y formas espaciales.

**Conclusiones:**

las formas espaciales trasladan el simbolismo al espacio social físico con características que afianzan el ejercicio de su saber y su identidad. La brecha entre los conocimientos occidentales y los saberes tradicionales se cierra cada vez más por la fuerza de sus realidades en la vida cotidiana, de una sociedad globalizada.

## Abstract (en)

**Introduction:**

revaluing and re-signifying the identity of traditional health knowledge is challenging in a culture mediated by industrialization, surviving with a historical past, cultural and symbolic heritage, from a mixed identity that is conjugated in a territorial syncretism, which persists through physical and cultural traces of magical-religious traditions.

**Objective:**

to recognize the ethnographic particularities of the agents who carry popular knowledge in health that allows them to strengthen their practices in the Andes-Antioquia municipality. To re-signify these practices to recover their identity and symbolic ritual environments, in the configuration of their identity as an organic body in social spaces.

**Methodology:**

qualitative, descriptive type research, with ethnographic perspective (micro-ethnographic), systematized through in-depth interviews, accompanied by photographic records and participant observations.

**Results:**

48 social agents between 30 and 70 years old, most of them settled in rural areas, were identified. Among them: sobanderos with secret, sobanderos with pain or componedores, hierbateros and rezanderos. Their diverse identity is recognized from a magical religious emergence and the characteristics of their inherited knowledge are exposed, in an enveloping rituality that recreates and vitalizes them in social spaces.

**Discussion:**

the results are analyzed within the framework of social, multidimensional and complex spaces, through its three elements: social structures, social relations and spatial forms.

**Conclusions:**

spatial forms transfer symbolism to the physical social space with characteristics that strengthen the exercise of their knowledge and identity. The gap between western knowledge and traditional knowledge is closing more and more due to the strength of its realities in the daily life of a globalized society.

## Resumo

**Introdução:**

revalorizar e resignificar a identidade dos conhecimentos tradicionais em saúde é um desafio em uma cultura mediada pela industrialização, sobrevivendo com um passado histórico, cultural e simbólico, a partir de uma identidade mista de sincretismo territorial, que persiste através de traços físicos e culturais de tradições mágico-religiosas.

**Objetivo:**

reconhecer as particularidades etnográficas dos agentes portadores de conhecimentos populares em saúde que lhes permitam fortalecer suas práticas no município de Andes, Antioquia. E resignificar estas práticas a fim de recuperar seus espaços de identidade e rituais simbólicos, na configuração de sua identidade como um corpo orgânico em espaços sociais.

**Metodologia:**

pesquisa qualitativa, descritiva, com uma perspectiva etnográfica (microetnográfica), sistematizada através de entrevistas em profundidade acompanhadas de registros fotográficos e observações dos participantes.

**Resultados:** Foram identificados 48 agentes sociais entre 30 e 70 anos de idade, a maioria deles instalados em áreas rurais. Entre eles: sobanderos com secreto, sobanderos com dor ou componedores, herboristas e curandeiros. Sua identidade diversificada é reconhecida a partir de uma emergência mágica religiosa e as características de seus conhecimentos herdados são expostas, em um ritual envolvente que os recria e os vitaliza nos espaços sociais.

**Discussão:**

os resultados são analisados no âmbito do espaço social, multidimensional e complexo, através de seus três elementos: estruturas sociais, relações sociais e formas espaciais.

**Conclusões:**

As formas espaciais traduzem o simbolismo em espaço social físico com características que fortalecem o exercício de seu conhecimento e identidade. A distância entre o conhecimento ocidental e o conhecimento tradicional está se fechando cada vez mais devido à força de suas realidades na vida cotidiana em uma sociedade globalizada.

## Introducción

La medicina tradicional colombiana es el sistema de curación que evolucionó desde la conquista europea, y se divide en dos ramas de acuerdo al origen de las enfermedades y las prácticas rituales de curación que aplican: el sistema del curanderismo y el sistema mágico-religioso. Ambas ramas, invocan espíritus y poderes para obtener la ayuda sobrenatural, y generalmente ambos atribuyen al dolor y al sufrimiento un origen punitivo proveniente de un ser supremo o fuerza sobrenatural. El sistema Mágico-religioso fundamenta la enfermedad y la cura en una fuerza sobrenatural, a través de un agente intermediario. El sistema del Curanderismo resulta de la asimilación-negociación entre prácticas curativas antiguas y la medicina occidental ^( 1)^.

Revalorizar y resignificar la identidad de estos saberes prácticos en el campo de la salud tradicional es importante y retador, en una cultura, en la cual han mediado procesos de colonización, comercialización, e industrialización, perviviendo con un pasado histórico, patrimonial cultural y simbólico. Estos saberes preservados desde una identidad mixturada con pueblos indígenas, y una tradición religiosa con características míticas, se conjugan en un sincretismo territorial, a través de huellas físicas y culturales de tradiciones médicas. De allí que sea importante su recuperación desde un enfoque cualitativo, plasmado desde sus voces. Este estudio reconoce la potencialidad que tiene la pervivencia de estos saberes en salud, en una época, la del mundo globalizado, que desde la realidad de sus prácticas posiciona unos agentes sociales provenientes de una realidad religiosa y sanitaria, que reta a su reconocimiento y agenciamiento social.

La colonización antioqueña creó un estilo de cultura entre las familias cafeteras, que se evidencia al recorrer el Municipio de Andes, el cual está ubicado en valle de San Juan en la región suroccidental del departamento de Antioquia, Colombia, en la vía troncal del café, la cual conforma con los municipios de Amagá, Ciudad Bolívar, Hispania y Jardín, generando un fuerte impacto comercial en la región y el país. Andes con sus 6 corregimientos divididos en 64 veredas y, 21 barrios en la cabecera municipal, cuenta además con un contexto cultural de fuerte arraigo y tradición religiosa, la cual comparte con sus municipios vecinos como Jericó y Jardín conformando una zona turística católica, Así mismo, este territorio alberga de manera focalizada una tradición indigenista, de cultura Embera Katio (2).

La fundación de los corregimientos en general se dio a partir del levantamiento de una iglesia central, alrededor de la cual se asentaron las casas y lugares públicos. *“Esta tipología de origen netamente religioso se ha visto marcada en toda la historia del corregimiento y es representada en la devoción católica evidente que ha sido profesada por la mayoría de habitantes, al igual que en la visión hacia los párrocos como líderes sociales”* (3). Muestra de este sincretismo, son las fiestas y eventos más representativos con fines religiosos y culturales, tal como las Fiestas Katias en honor a las comunidades indígenas en el municipio.

A través de los recorridos, actividades y entrevistas realizados en el municipio en el marco de las prácticas académicas desde el 2013 hasta el 2019 de la Universidad de Antioquia (3,4,5,6,7), se pudo reconocer la presencia de agentes portadores de saberes tradicionales de raigambre popular entre sus pobladores, las cuales se mantienen a través de diversas prácticas de cuidado y de atención en salud, y que son además acogidas por los habitantes del municipio.

Para la corriente estructuralista de la sociología, estos portadores de saberes son *agentes sociales,* ya que hacen uso de ellos y son productores de estos mismos ya que tienen una especial preocupación por su transmisión y permanencia en la cultura. Dichas prácticas y saberes a su vez están condicionadas por el espacio donde se desarrollan. Como lo expresa Pierre Bourdieu (8), los agentes son “*el producto de la historia, esto es, de la historia de todo el campo social y de la experiencia acumulada en el curso de una trayectoria […] los agentes sociales determinan activamente, mediante categorías de percepción y apreciación social e históricamente constituidas, las situaciones que los determinan”.*

Lopes da Silva(9) reconoce que *“los saberes populares surgen de diversas experiencias de vida y formas de conocer el mundo que se producen, y son heredados o tienen su origen en los medios populares, en los movimientos sociales y/o en los ámbitos religiosos, étnicos, asociativos con intenciones de ciudadanía, de resistencia cultural o de negociaciones simbólicas”.* Se trata de saberes que contribuyen al desarrollo de todas las potencialidades y dimensiones del ser humano (biopsicosociales, generacionales, de género, de etnia, de relación con lo sagrado, etc.). Y que, si bien se producen desde la experiencia individual de cada persona, *“sumadas en colectividades dan forma a distintas identidades.”* Así mismo, para Vila(1) la medicina tradicional, además de sus elementos prácticos y teóricos (proveniente de los saberes populares), debe cumplir con poseer un arraigo histórico, cultural y social, en el entramado de la historia de un pueblo, conformando un sistema más formal de conocimiento.

De allí que el presente estudio se pregunte por las particularidades etnográficas de estos agentes y las características que les permiten afianzar sus prácticas de tradición médica en el municipio. Así mismo la pregunta por: ¿cómo es posible resignificarlas para reconocerlas?, para recuperar sus entornos identitarios y rituales simbólicos, en la configuración de su identidad como cuerpo orgánico en los espacios sociales.

## Materiales y Métodos

Esta investigación cualitativa, de tipo descriptivo, inscrita en la perspectiva etnográfica, de tipo micro etnográfica. Hace referencia a *“...la descripción (grafé) del estilo de vida de un grupo de personas, habituadas a vivir juntas (ethnos)*”, En ésta perspectiva los agentes sociales cumplen un papel protagónico, haciendo uso de la descripción como elemento fundamental para comprender y caracterizar estos fenómenos sociales(10). Los agentes sociales se convierten entonces en *“informantes privilegiados pues sólo ellos pueden dar cuenta de lo que piensan, dicen y hacen con respecto a los eventos que los involucran”*(11)*.* Quienes actuaron en calidad de investigadores y coinvestigadores acompañaron todo este proceso reflexivo intencionando sus búsquedas, a través de un proceso de comprensión e interpretación, que tuvo la particularidad de conservar en sus relatos, los sentipensares de los protagonistas, desde su realidad presente y su expectativa futura, en consenso con los procesos interpretativos de los investigadores.

La población sujeto de estudio estuvo constituida por personas reconocidas y caracterizadas socialmente en el municipio de Andes como agentes portadores de prácticas y saberes en el campo de la salud, en el espacio urbano y rural. Se reconocieron a través del trabajo de campo 60 agentes, de los cuales se tuvo contacto directo con 48. Sus edades oscilaron entre 30 y 70 años, en su mayoría asentados en la ruralidad. Es de notar que algunos de ellos son reconocidos por las instituciones de salud local y han afianzado sus saberes a través de procesos de capacitación impartidos por estas. Se abordaron en sus espacios naturales cotidianos (casa o sitio de trabajo) donde desarrollaban las actividades propias de sus saberes. Esto para propiciar un encuentro de mayor confianza y obtener una descripción lo más ajustada a la realidad.

La selección de los agentes se dio a partir de un proceso progresivo (Estrategia de bola de nieve), sujeto a la dinámica de su reconocimiento en los relatos de otros agentes, permitiendo la agregación de nuevos informantes. La muestra inicial se pudo reconocer a partir de la matriz de caracterización propuesta por Vila(1) y ajustada en el estudio, de acuerdo a las condiciones y dinámicas del trabajo de campo y la realidad de los agentes. En correspondencia con la tradición de carácter cualitativo, la constitución de la muestra se dio desde el cumplimiento de los siguientes criterios de inclusión:

1. Ser reconocidos como agentes portadores y reproductores de saberes por parte de la comunidad.
2. Que al momento del contacto con los investigadores realizaran y fundamentaran sus prácticas.
3. Que se reconociera en ellos una tradición en la transmisión de sus saberes.

La recolección de la información se dio a través de una caja de herramientas, que incluyó entrevistas a profundidad, las cuales se orientaron con preguntas desencadenantes acerca de su constitución como agentes portadores de un saber a manera de relatos de vida, acompañadas de registros fotográficos en sus espacios sociales; además de una constante observación participante durante todo el trabajo de campo por parte de los investigadores, los cuales consignaban sus hallazgos en cuadernos de notas, a manera de relatos.

El trabajo de campo se inició en el año 2018 y se cerró en el 2019 a partir de la saturación de la información que se presentó en los datos recolectados, a partir de las constantes identificadas en cada una de las categorías emergentes que se hallaron. En esta actividad de saturación cumplió un papel importante la triangulación de la información, procedimiento práctico que se sirvió de la siguiente secuencia: 1. Se seleccionó la información obtenida bajo criterios de pertinencia, tomando la información relevante desde su recurrencia y asertividad. 2. Se trianguló la información recolectada a partir de un proceso inferencial que consistió en establecer conclusiones ascendentes, agrupando las respuestas relevantes por tendencias, que fueron clasificadas en términos de coincidencias o divergencias, en cada uno de los instrumentos aplicados. En este proceso emergieron varios niveles de síntesis desde los relatos inferidos en relación con las preguntas que guiaron la investigación. 3. Finalmente se cruzaron los resultados obtenidos agrupándolas por categorías, confrontándolas con las teorías socialmente existentes (reducción teórica).^1^

El estudio contó con el aval del Comité de Bioética de la Facultad de Odontología de la Universidad de Antioquia, según consta en el Acta institucional del Centro de Investigaciones en el Acta N° 01-2017. Fueron considerados los aspectos éticos acorde a la Resolución 8430 de 1993 del Ministerio de Salud y Protección Social de Colombia, considerándose una investigación “de riesgo mínimo”. Además, se realizó un consentimiento informado con cada uno de los participantes teniendo en cuenta la confidencialidad de la identidad de los agentes que participaron voluntariamente en el estudio.

## Resultados

### Identidad Diversa, una Emergencia Mágico-Religiosa en los Saberes Tradicionales en Salud

Los agentes portadores de saberes populares en salud se agruparon, como ya se había mencionado, según la matriz de clasificación propuesta por Vila(1), acorde a las características de sus prácticas en las expresiones de las ramas de la medicina tradicional: sobanderos con secreto, sobanderos con dolor o componedores, hierbateros y rezanderos, los cuales se encuentran asociados a un saber mágico-religioso. Para Castilla(12), en una era de globalización, la búsqueda del sentido espiritual como respuesta a la búsqueda de la salud, pudiese estar relacionado con la crisis de credibilidad de la institución médica o religiosa, dos instituciones que cuya crisis producto de una sociedad neoliberal, de competencia de mercados y de un sincretismo religioso, dejaron un hueco en los ámbitos eclesiásticos y sanitarios donde tendrían sentido y cabida las medicinas tradicionales y toda una serie de rituales de protección, como una alternativa para la búsqueda de una terapéutica o creencia determinada, que en definitiva son el resultado de dos caminos el simbólico y el técnico, y dos conceptos -la religión y la medicina- que no se excluyen, y son complementarios en la búsqueda de la salud integral.

En el ámbito urbano del municipio de Andes se reconoce una presencia significativa de agentes rezanderos y sobanderos con secreto, y en el ámbito rural los hierbateros y sobanderos/ componedores^2^. En este sistema cultural alrededor de sus creencias y saberes se dan respuestas de orden empírico y de carácter sobrenatural, desde un orden metafísico, se busca por tanto una acción curativa, de un modelo religioso, el cual se reviste de un poder sanador afianzado en y a partir de un instrumento simbólico, que es catalizado hacia una ritualización que soporta la creencia; lo que representa para Ordóñez(13) “*un conjunto de procedimientos donde lo parecidoprovocaloparecido, la parte representa al todoyunefímerocontactoocontigüidadconecta mágicamente objetos y pensamientos”.*

Todo el sistema de medicina tradicional lleva aparejado un ritual para invocar la participación de los sujetos sobrenaturales en la reordenación de un hecho, la salud y la desaparición de la enfermedad o sus manifestaciones cercanas; inicia por tanto la evocación al acto ritual, el cual continúa con una invocación de dicho ser sobrenatural, que se acompaña de objetos, expresiones sígnicas, como las cruces en el caso del sobandero con secreto, las expresiones físicas, como la forma en que se dispone la ceniza de un tabaco, o los círculos de residuos en las tazas de un rezandero, así mismo la acción mecánica en el sobandero, o la forma en que se preserva o dispone una planta, en un yerbatero. Es una magia que enlaza actos con motivos, con medios, en un procedimiento mental al que se le otorga una fuerza y eficacia, en las operaciones mágicas, en las representaciones, pensamientos y deseos donde se desplazan y condensan, en un fundamento psicológico para esas creencias y prácticas culturales(13).

### La fe, una tradición mediadora en los saberes populares en salud

El surgimiento de éstas prácticas en el modelo mágico-religioso provienen al igual que las doctrinas, como fenómeno humano, de dos tipos de explicaciones: la primera como forma de satisfacción de la necesidad teórica para explicar o detener un conocimiento y la segunda de una necesidad práctica conducente a una solución; ambas bañadas por el temor que produce en el hombre la incertidumbre que se quiere o puede asegurarse por la presencia de un ser superior, el mal, al cual se teme y la seguridad frente al bien, al tiempo que se logra un conocimiento. De ahí que algunos provengan de la tradición de fuentes primarias orales, de nexos cercanos como los familiares, o de tradiciones religiosas, o por el interés propio. Priman en ellos el ejercicio práctico, los preceptos y conceptos, los cuales son asegurados por la intuición y en algunos de ellos por el respaldo teórico de textos guardados con sigilo alrededor de altares o dispensarios.

La palabra “fe” viene del griego *pistis*, denota una creencia determinada por confianza (o seguridad) predominante, en un Dios, que surge de la fe en los mismos. La religión cristiana la define como la certeza de lo que se espera, la convicción de lo que no se “ve”, fe es la esencia o certidumbre de algo que esperamos y no hemos recibido. Como ya se ha descrito, en el marco del arraigo religioso propio del municipio de Andes, es notoria la presencia de objetos e imágenes religiosas (Ver Imagen 1), específicamente católicas en los hogares de todos los entrevistados.

En particular pudieron observarse objetos y símbolos de este tipo en las viviendas de los agentes sobanderos con secreto y curanderos. El poder sanador es atribuido a Dios, quien actúa a través de un mediador poseedor de un “don”, en este caso el agente, y la fe de la persona que se acerca con el fin de obtener pronta cura de su dolencia o situación de salud. Los agentes curanderos rezanderos ponen su fe en su capacidad de adivinación y en los objetos o elementos rituales usados, relatando que:

*“Por ejemplo, si llega una persona que no cree en Dios, no funciona... si usted va a tomar cualquier medicina sea lo que sea: señor haz que esta medicina que me voy a tomar sea en el nombre tuyo la curación para mi enfermedad” MRH.EC*

*“Tiene que tener fe el que se le va a hacer y que uno también lo tiene que hacer con fe. Yo les digo -no es coger esta oración, leer... es concentrarse en la persona y hacerla, porque si usted va a leer como loca, a usted no le va a servir” RH.MLR*

Los agentes hierbateros, además ponen su fe en las propiedades y dones de las plantas, razón por la cual, hacen ahínco en el rito de preparación y en las partes de la planta usadas:

“.*..trabajo mucho la zanahoria, en la zanahoria encuentro yo muchas formas para ayudar a las personas, tiene demasiadas vitaminas, elementos, para muchas clases de enfermedades, principiando porque la zanahoria da mucho oxígeno… “MH. LA*

**Imagen 1:**
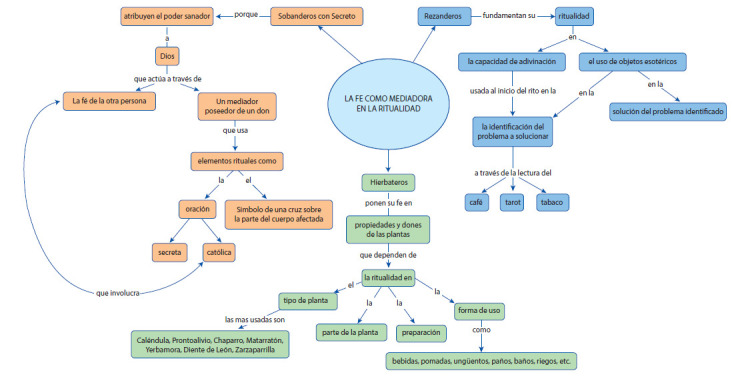
La fe. Autoría propia.

### Saberes heredados, intuición - aprehensión, y dones.

La manera en que los agentes adquieren los saberes e inician la práctica en salud es diferente. Según un estudio realizado en Perú(14), los agentes sociales adquieren o se inician en sus saberes en medicina tradicional a través de: la selección natural/mítica, que implica una manifestación hecha por seres superiores que se manifiestan, por ejemplo, a través de sueños, la caída de un rayo u otro fenómeno extraordinario; a partir de un maestro o mentor que puede ser un familiar o no; y a partir de la experiencia, observación y reflexión propia. Sin embargo, se encontró como elemento común entre los agentes entrevistados que la mayoría de ellos refería la inexistencia de un aprendiz que hiciera perdurar sus saberes y prácticas, por un desinterés de la nueva generación en la pervivencia y permanencia de dichas tradiciones. Los sobanderos con secreto reconocen en la adquisición del saber e inicio de las prácticas cierto misticismo (Ver Imagen 2), ya que reciben de alguien una oración, las cuales sólo deben ser conocidas y recitadas por ellos mismos, ya que se hereda a condición de no ser divulgada sino transmitida a otra persona cada siete años y usada para el servicio a los demás sin exigir una retribución económica, como lo relata:

*“El año pasado le di a un sobrino mío… se la di porque es buena gente, es responsable porque uno entregarle la oración a cualquier persona por ahí que uno no sepa quién es ni qué quiere hacer con ella ahí sí no” M.RE*

Teóricamente, la intuición es entendida por Builes(15) como*“ la capacidad de síntesis de muchos elementos”* percibidos en la experiencia subjetiva, en la que “*lo percibido cobra el carácter de «verdad» y se toma por cierto, y por tanto no es corregible por la acción de la razón; y permite captar aspectos esenciales o constantes de los objetos (Lorenz, 1993)”*; esta autora menciona en otra de sus obras que, históricamente la intuición se ha reconocido desde dos perspectivas: una teológica, relacionada a la bienaventuranza dada por Dios, y otra de carácter filosófico, asociada a la captación de datos mediante la percepción o a las ideas puras que surgen a partir de la razón. Además, la aprehensión implica. o comprensión de una idea o un conocimiento por completo, un arraigo de los saberes en el imaginario.

De manera complementaria, para acercarse al concepto “don”, el trabajo de Diaz(16) menciona a la omnipotencia de las ideas como un fenómeno extraño del poder *“que emana de la presencia del curador, (...)Es un poder mágico que tiene dos polos: el del sanador que desea sanar y el del enfermo que desea ser sanado*. En este estudio se encuentra que algunos agentes se refieren al origen de su saber cómo algo que nació con ellos, que se fue manifestando de forma intuitiva en su vida por medio de situaciones en las cuales han adquirido experiencia y a causas místicas o sobrenaturales siendo el poder de curar al otro, un don dado por Dios, como en el caso de algunos sobanderos/componedores:

*“Mire, para uno que es católico, eso es don de Dios, no sé explicarle, cómo nació en mí eso, simplemente, veía a la persona que caía… me nació, intuitivamente, sabía yo qué tenía que hacer” SJR*

Los agentes hierbateros se apoyan y fundamentan su práctica en la lectura de libros de tipo gnóstico que tienen contenidos de botánica como el referido “Magia blanca”, y libros sobre la clasificación de las plantas y sus usos para el alivio de síntomas y tratamiento de enfermedades, los cuales fueron encontrados en tiendas naturistas del municipio como el libro llamado “Las Tradiciones de los Abuelos”. Otro libro mencionado fue “Magia tráfica”. Además, son frecuentemente consultados libros de oraciones, novenas, etc., de contenido religioso.

De otro lado, el origen de las oraciones que practican, los agentes rezanderos pueden ser de tipo religioso católico o esotérico, y algunos agentes utilizan ambas ya que fundamentan su saber en ritos, que integran oraciones y deidades católicas y de otras fuentes entre las que resaltan conocimientos acerca de las energías, de magia blanca y gnosticismo. Estos agentes relataban en general que su capacidad de adivinación y curación proviene de un don o de la acumulación de experiencias empíricas en esta labor. Por ejemplo, un agente relata: *“Un señor me vio mal con mis hijos y me enseñó lo que él sabía hacer, leer el tabaco y hacer amarres” RH. MLR*

**Imagen 2:**
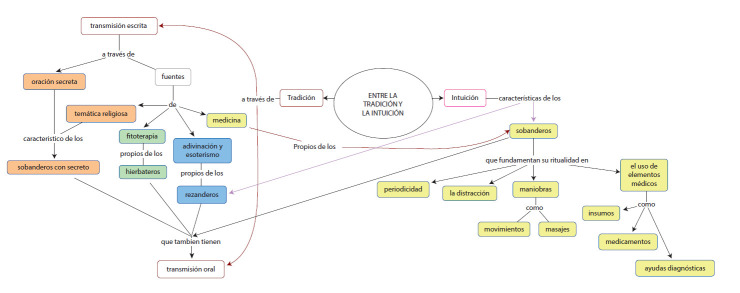
La fe. Herencia e intuición. Autoría propia.

### Práctica tradicional y la ritualidad.

Las prácticas de la medicina tradicional se caracterizan por la ritualidad. El rito viene a ser en la práctica un *“mecanismo simbólico de la vida social… que contribuye a la regeneración permanente o periódica de esa vida, a lo largo de las generaciones, mediante su repetición”.* Las acciones rituales son elaboradas, ya que generan una articulación de elementos diversos como gestos, cantos, rezos, palabras, que se dan en lugares determinados a ese fin “*utilizando objetos y parafernalias a veces muy sofisticados. Se trata de una actuación pre programada, estereotipada y codificada”*(17)*.*

Los agentes sobanderos con secreto evidencian la ritualidad de su práctica en el uso de oraciones como elemento primario que pueden complementarse con elementos religiosos, tales como el trazo del símbolo de la cruz con el dedo, el rosario, la oración a la Santísima Trinidad, y oraciones como el padre nuestro y el credo:

*“Por ejemplo tiene un golpe aquí (señala el dorso de su mano), pongamos que están hinchados, entonces yo le cojo la mano pacito, y le digo ¿dónde le duele?... entonces pongo la mano mía encima, le rezo mientras hago las crucecitas y eso es todo” M.RE*

Especialmente el sistema del Curanderismo tiene su fundamento en la ritualidad y utilización de utensilios o elementos rituales. Los sobanderos/componedores, por ejemplo, utilizan diversos insumos de tipo médico. El ritual da inicio con el señalamiento de la ubicación corporal de la molestia o dolor, en muchas ocasiones el agente le pide a las personas que se tomen algún tipo de ayuda diagnóstica como un radiografía o utiliza las ya tomadas en los servicios de salud, el agente mientras distrae a la persona con conversaciones, comienza a realizar movimientos de extremidades y articulaciones masajes o presión en los lugares indicados según sus conocimientos hasta lograr posicionar los huesos y músculos adecuadamente con un movimiento rápido. Ello implica dolor y por lo general hay inflamación, por lo cual recetan o ellos mismos aplican analgésicos antiinflamatorios.

*“Por ejemplo, la cuerda está acá (en la mano), se zafó acá, se empieza a sobar (en círculos) con cremita caliente, así, sobar, sobar y calentar, ya apenas esté bien caliente ya uno (mueve la mano hacia arriba) y ya, ahí mismo traquea y metió el hueso, pero uno tiene que saber, porque si es fractura, uno toca, y el huesito, la astillita, lo choca uno, por eso uno no se puede meter, yo cuando siento algo raro le digo, “hágase una radiografía” y viene… cuando es descomposturas, se soba una vez o tres, porque mamá decía que si usted sobaba dos veces el hueso no queda bien, tiene que ser una o tres” S.MEM*

Los agentes rezanderos al inicio del ritual se esmeran en conocer muy bien la situación que está atravesando la persona que acude a ellos, con el objetivo de definir qué rezo o tipo elemento ritual es el indicado para solucionar su problema, el cual puede no ser solo de tipo físico, sino emocional o relacional. Algunos agentes también refieren que las necesidades de sus consultantes pueden ser de apoyo emocional y espiritual. Luego realizan una oración o el ritual que requiera:

*“Empiezo a leer el tabaco y les voy diciendo lo que sale, cuando tienen un maleficio o no lo tienen, cuando hay enfermedad. Leo la ceniza, la ceniza que yo voy fumando...y ya un amarre es con aguacate, hay que abrirlo en dos mitades poner el nombre, hacer dos corazones echarle la esencia de esa de amarre que venden en las cosas esotéricas, que es para atajar el marido o la mujer, amarrarle una cinta roja y alumbrar con 3 veladoras, una vela por día. Y ya ahí está el amarre.” RH.MLR*

Se observa que en la práctica de los curanderos hierbateros el ritual se expresa tanto en la forma de preparación como de uso de la planta usadas para la elaboración de bebidas, pomadas, ungüentos, paños, baños, riegos, etc. en los otros elementos que componen su preparación, en el tiempo que dura el tratamiento y demás instrucciones. Las indicaciones van desde dónde puede conseguir la planta, la parte de la planta que se usa, y de qué manera se debe tomar o aplicar:

*“Hay un palo que se llama Chaparro, es muy bueno… coge uno un palito de unos quince centímetros, si es delgadito; si es grueso más pequeño, pero tiene que ser seco, porque verde no sirve, usted lo pica chiquitico y lo echa en dos litros de agua y lo deja remojar cinco o seis horas. A las cinco o seis horas él suelta una cosa como negra, lo pone a hervir… las bebidas son nueve bebidas, si a usted una persona le dice: tómese unas bebidas de tal planta, no vaya a dejar de tomarla... si deja de tomarse una ya no, ya no sirve” MRH.EC*

Se obtuvo un inventario de 53 plantas, con su uso y forma de administración. Algunas de las plantas más mencionadas por los hierbateros en Andes son aquellas que tienen propiedades antiinflamatorias y analgésicas como Caléndula, Prontoalivio, Altamisa, Penca Sábila, Manzanilla, y Guayaquil. Para los problemas respiratorios se da uso a plantas como Eucalipto, Sauco, Orégano, Hierbabuena y Jengibre. Para los problemas gastrointestinales se utilizan la Alcachofa, Masequía, Apio, Jengibre, Verdolaga, Limoncillo, Boldo y Flor de Jamaica. Todas ellas se administran de manera oral bebiendo infusiones realizadas al hervir o dejar reposar en agua las hojas o flores de la planta. Otras plantas de uso tópico se maceran y aplican sobre la piel como antiinflamatorios son la caléndula, la hoja de plátano y el romero. Algunas plantas tienen usos de adivinación o esoterismo, por ejemplo, la llamada destrancadera, abrecaminos, y ruda a la que confieren buena suerte, y se usan para realizar baños a las personas o riegos a los espacios con su infusión.

### Lo mágico religioso de la salud y enfermedad en el saber tradicional.

En cuanto a la manera en que definen el proceso de la vida, la salud y la enfermedad se hace evidente la concepción religiosa arraigada a su cotidianidad y cultura. En manifiestas ocasiones los agentes afirman que la vida es un regalo ofrecido por Dios. Así mismo la salud viene de él, como creador supremo y proveedor de todo, de esta manera comprenden que la enfermedad, lo patológico, se convierte en un reto o falta de espiritualidad, el acto de alejamiento a Dios, siendo la enfermedad incluso una prueba o castigo:

*“La enfermedad… no sé de dónde proviene, porque considero que son cosas de Dios, él nos las pone como una prueba para que nosotros reflexionemos, sigamos adelante, y que, así como él sufrió tanto nosotros tenemos que pagar con las enfermedades.” M.RE*

Los agentes reconocen que algunas enfermedades deben ser tratadas por médicos ya que son “dolencias físicas”, diferentes de las “enfermedades espirituales”, como relata uno de ellos: *“... hay mucha gente a veces acude donde nosotros, y yo les digo “¿ya fueron a un médico?, ¿ya le hicieron estos exámenes?… porque es que de pronto es una enfermedad lo que tiene la persona y no es algo espiritual*” R.MM

En ese sentido, Vidarrue (2006) es citado por Laza(18), para conceptualizar la salud “*como un equilibrio armónico y dinámico entre el cuerpo, la mente y el entorno social y natural del individuo. De tal manera, si existiese una trasgresión a este orden natural se estaría desequilibrando la homeostasis del sistema; este desequilibrio se denomina enfermedad; identificándose la existencia de una relación muy fuerte entre cuerpo, espíritu y alma, pero no como realidades divididas, más bien formando parte del mismo tejido celular*”.

### Identidades y espacios sociales que recrean las prácticas de medicina tradicional en el territorio^3^

Para Santos(19) el espacio se define: *“como un conjunto de formas representativas de las relaciones del pasado, y del presente y una estructura representada por las relaciones sociales que ocurren ante nuestros ojos y que se manifiestan por medio de los procesos y las funciones”,* de tal forma que el espacio se representa de manera multidimensionales en tres elementos claves, formas espaciales, relaciones sociales y estructuras sociales.

Las formas espaciales contenidas y distribuidas se concretan en un espacio habitado, cargado de simbolismos. En los **Sobandero con Secreto,** una representación espacial que puede ser relatada como el de una casa antigua, contenida en una decoración igualmente antigua de una gran sala que conecta con una pequeña habitación y la cocina, la sala en su interior con dos muebles, una cama, sillas y mesas de madera sobre las que se posan el televisor, el equipo de sonido y otros objetos como lámparas y floreros, se encuentran algunas plantas naturales ornamentales. Las paredes blancas estaban decoradas con muchos cuadros de tipo religioso como imágenes de Jesús, La Santísima Trinidad, la Virgen María y los ángeles; se destaca la imagen del Sagrado Corazón de Jesús ya que es el cuadro más grande y se encuentra en el centro de la pared de enfrente, justo arriba del televisor.

A la derecha del marco que da a la cocina hay un rincón decorado de forma particular, que conforma un pequeño altar con algunas flores artificiales, compuesto por un conjunto de objetos religiosos colgados en la pared como un pesebre, cruz de madera, camándula, estampitas con oraciones, imágenes de la virgen, ángeles y el Divino Niño, y cuadro de Jesús con la frase “yo soy el camino, la verdad y la vida”. También se aprecia un escaparate de madera, lleno de estatuas de esas mismas deidades y al lado una biblia abierta encima de una silla pequeña. Muchos de los objetos de tipo religioso observados en la casa como las estatuas e imágenes son regalos que las personas le entregan en agradecimiento por su labor como sobandera. En esa misma sala, en la pared junto a la cama entre objetos decorativos como fotos familiares, relojes, llaves, herradura, jarras y porcelanas, hay colgada otra figura del Niño Jesús, de un ángel y un cuadro de San Juan Bosco.

En la habitación, separada por una cortina blanca de la sala, se observan cuadros con fotografías a un costado. En la habitación del lavado y la cocina hay una mesa-comedor pequeña y un escaparate con productos y artículos de cocina, tiene encima de un muro en la cocina, macetas con penca sábila y cebolla de rama. Además de imágenes, es frecuente observar que algunos de estos agentes escuchan emisoras radiales de oración, lo cual genera un ambiente propicio de religiosidad y característico de un espacio ritual, permitiendo realizar una lectura del grado de espiritualidad, devoción y creencia religiosa de dichos agentes.

**Imagen 3:**
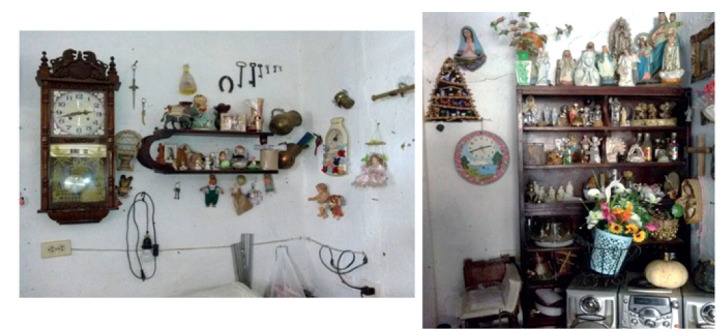
Espacio e identidad de Sobandero con secreto. Autoría propia.

Una agente Hierbatera y Rezandera, vive con dos hijos y una hija en una casa pequeña y adyacente a una quebrada en la zona urbana de Andes. La sala es estrecha y sirve como sala de espera para para aquellos que buscan su atención, en este espacio hay algunos cuadros ornamentales y fotografías familiares, además resalta un manojo de penca sábila amarrada y colgada junto a la puerta. Al lado de la sala está el comedor, lugar en el cual la agente recibe a sus clientes y realiza la lectura de tabaco, encima de la mesa se encuentra un velón negro de 7 mechas en su empaque con un papel que tenía por título “Oración tumba trabajos 7 mechas”, también un recipiente con medicamentos y con insumos como bolsas plásticas y de telas. Se nota un empaque de productos naturistas de la planta Caléndula. Al lado del comedor se encuentra una silla de plástico roja que la agente usa para sentarse mientras atiende a las personas.

Junto al marco de la puerta se puede ver una cruz de madera colgada y una repisa decorativa con una planta Quiebrabarriga. En esta misma zona de la casa, en una de las repisas sobre la lavadora se encuentran algunos productos como lociones y esencias de tipo esotéricas entre las cuales se identifican algunas usadas para atraer pareja, para la abundancia, y la buena suerte. Sobre la lavadora se encuentran los tabacos en su caja junto a las cerillas y un cenicero de plástico rojo con ceniza en su interior. Junto a la lavadora se observa una abertura en la pared que permite la salida del humo de tabaco de la casa con un muro de altura mediana sobre el cual se observan algunas macetas con plantas ornamentales y medicinales como la Moringa y el Pronto Alivio. En este espacio del comedor se puede identificar un cuadro del Sagrado Corazón de Jesús y una estampa de la virgen María colgados en la pared al frente del comedor.

**Imagen 4:**
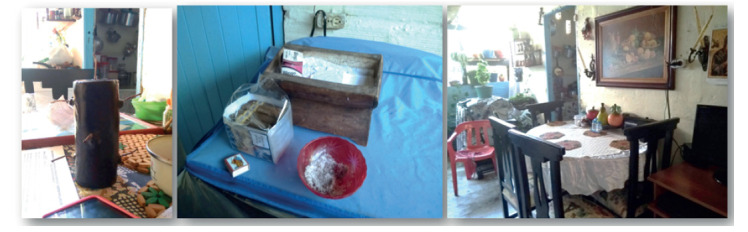
Espacio e identidad de Rezandero y Hierbatero. Autoría propia.

De otro lado la casa del agente Sobandero/Componedor, es el segundo nivel de una vivienda antigua en la que ha vivido toda su vida, ubicada en la cabecera municipal. En las paredes de la sala se pueden identificar gran cantidad de objetos religiosos como una cruz de madera y un pesebre pequeño puesto sobre una repisa de madera, un cuadro del Sagrado Corazón de Jesús y otros elementos antiguos como una radio de gran tamaño y un televisor de perilla, cassettes, cuadros decorativos, libros y un trofeo.

Su habitación, contigua a la sala, es pequeña y sobre su cama se encuentra posada una porcelana del Divino Niño Jesús, en la cabecera se aprecia un cuadro de la Virgen María. En el corredor se encuentran dispuestas algunas macetas con plantas ornamentales y hay colgado en la pared un cuadro de la Última Cena justo arriba de un bifé que tiene objetos decorativos y un velón blanco mediano. La cocina queda al final del corredor, en ella se observa una mesa tipo comedor de madera con sillas, lugar en el que el agente atiende a las personas que acuden a él. Sobre el comedor hay dos grabadoras y muchos de los implementos que el mismo agente compra para usar en su práctica, como son jeringas desechables, vendas, algodón, hielo mineral, gasas, Betametasona en crema y ampolla, Vic Vaporub, tabletas de Acetaminofén e Ibuprofeno. También se observa encima de la nevera ubicada en este mismo espacio insumos como alcohol antiséptico y pomada caliente. Estos implementos son almacenados en varios cajones ubicados en el bifé de madera al frente del comedor.

**Imagen 5:**
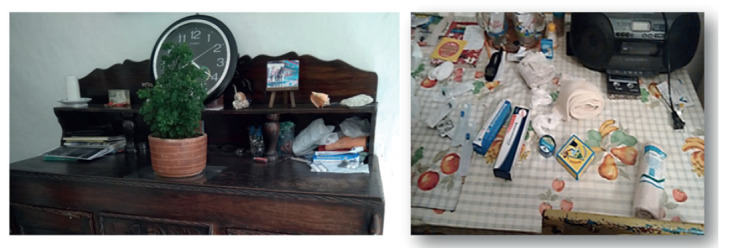
Espacio e identidad de Sobandero con dolor o Componedor. Autoría propia.

Estos espacios se configuran en espacios sociales de práctica, algunos agentes realizan su práctica exclusiva o preferentemente en sus casas en un espacio específico destinado para tal fin, como el comedor, o la sala de estar. Otros agentes en cambio atienden a las personas que buscan su ayuda en cualquier lugar, pero siempre llevan implícita la carga simbólica de sus creencias.

*“Yo más que todo aquí (en el comedor de su vivienda), tiene que ser algún caso, que no se pueda mover, para que no me gusta hacerlo así, para que yo aquí, tengo de todo, los geles, el alcohol, el algodón, las vendas…” S.JR*

## Discusión

La estructura social, como elemento del espacio social, se refiere al sustrato de la vida social que se da en el orden inconsciente que subyace en la cotidianidad. Dicha cotidianidad se manifiesta en diferentes relaciones, saberes, poderes, y por supuesto, prácticas(18). Las prácticas populares en salud permiten a las personas una relación especial con lo sacro, con lo divino, con lo mágico, como alternativa ante la angustia que genera la debilidad, la enfermedad o el accidente, *“dirigiéndose a los seres sagrados mediante un procedimiento conocido para que éstos participen en la solución del problema. Ese proceder, que a simple vista puede suponer una incoherencia con otros sistemas de conocimiento y otros procedimientos de actuación, sean religioso o biomédicos, no parece suponer un problema para los que lo practican”*(12)*.*

En ese sentido, parece que hay un juego complejo, en el cual, los eventos y circunstancias de la vida no son producto del azar y ni siquiera de las voluntades personales y decisiones humanas, sino que son planeados por Dios. Pero donde, las personas no tienen que esperar a que la divinidad actúe por sí sola, *“sino que se le da la posibilidad de intervenir haciéndose partícipe del proceso. Se trata así de un sujeto que intenta convencer mediante el ruego, la plegaria o el sacrificio a una serie de seres capaces de lograr una solución inmediata”*(13). Por lo tanto, permite la acción, la fe en acción y ofrece esperanza al individuo.

Al igual que los agentes sociales de Andes, según los testimonios recogidos en otros estudios(14), la mayoría de terapeutas aprendió su labor siendo jóvenes y pocos tuvieron un maestro o mentor, o contaron con un terapeuta tradicional en su familia. La mayoría declaró haber aprendido ante la necesidad, al encontrarse con familiares o personas cercanas con alguna enfermedad o padecimiento, y muchos terapeutas refieren haber aprendido sobre la base de la práctica, recibiendo consejos, además de haber probado la efectividad de su repertorio terapéutico sobre la base del ensayo y error, así como por los resultados obtenidos en sus pacientes. Pero al igual que los entrevistados en dicho estudio, la mayoría de los entrevistados indicaron que no existen candidatos a aprendices. “*Explican que las generaciones más jóvenes no tienen interés en aprender, ni le dan la importancia debida*. También señaló que, aunque así lo quisieran, no disponen del tiempo ni los recursos suficientes para enseñar sus conocimientos a otra persona”.

Podría decirse que en medio de la de la ritualidad se genera un entramado de relaciones sociales, a partir de la realidad simbolizada desde los intercambios sanadores. Estos ritos, están relacionados respectivamente con acontecimientos de la vida comunitaria o la vida del individuo; y son de tipo ocasional, ya que la celebración se produce con motivo de acontecimientos cuya recurrencia no se da en plazo predecible; y, son primordialmente algo actuado, es práctica que implica una secuencia de actos cargados de simbolismo culturalmente codificado siendo más significativo lo que no se dice que lo que se dice(17).

En la ritualidad, un elemento privilegiado, son los amuletos, que son *“objetos cargados de magia tras ser sometidos a procedimientos y rezos rituales”.* Estos procedimientos siguen fórmulas de las artes mágicas, pero también de los actos religiosos entran en diálogo el cristianismo con la brujería. En este imaginario, los objetos son representantes de sujetos, fuerzas u otros objetos simbólicos, cómo las estampas, oraciones, imágenes, porcelanas, cruces. Estos objetos que se convierten en amuletos son un punto de contacto con las fuerzas espirituales del mundo sobrenatural y tienen el poder de proteger a sus dueños, y generar además un ambiente sacramental. Según Ordoñez, *“esta operación mental fetichiza el objeto, lo carga de representaciones simbólicas que se corresponden con las necesidades psicológicas de su poseedor”*(13)*.*

Como afirma Vila(1), estas prácticas y saberes tradicionales, aunque se intentan agrupar, clasificar y separar, no se pueden describir como un sistema o cuerpo homogéneo de rituales y agentes, sino más bien como un espacio de acción articulado y complejo, que se nutren de diferentes puntos de vista, y donde existen tensiones y juegos de poder. Ello se evidencia en los diversos nombres que reciben los agentes sociales según las características de la práctica popular en salud que realizan, por ejemplo: curanderos, sobanderos, componedores, hierbateros, etc. Existe una extensa diversidad de saberes y prácticas en salud que se pueden apreciar en el municipio de Andes, cada agente construye su quehacer en una amalgama compleja de estos saberes y prácticas populares que se entremezclan, ya que los agentes sociales se sirven de ritos y elementos que combinan la ritualidad de un sistema con otro. Se genera un sincretismo alrededor de los mismos, debido a que no se encontró una pureza en los sistemas de la medicina tradicional y sus expresiones.

Para Santos(18), las formas espaciales, es decir, lo visible y tangible, ganan presencia en los espacios donde moran las prácticas, la realidad habitada, donde se traslada y enriquece todo este simbolismo a un espacio de acción físico, con determinadas características y disposición de objetos que sirven para el ejercicio de ese saber. Ya que la composición de dichas formas espaciales es el resultado de las relaciones sociales que se dan en el mismo, relaciones de sincretismo, contradicción, convergencia y divergencia. El espacio es resultado de la forma en que intervienen los diferentes procesos y sus agentes, y cambia a su vez con las dinámicas sociales de los mismos. Los agentes le dan una explicación al uso y disposición de objetos que afianzan su identidad*.* Ello permite que se reproduzcan los saberes y prácticas populares y se concreten en la presencia y disposición de elementos que le dan sentido a las mismas y que, de esta manera puedan permanecer en la tradición.

## Conclusiones

El sincretismo recreado en los saberes populares en salud en el municipio de Andes, es el resultado histórico y social de unas estructuras y dinámicas sociales, relacionales, que los agentes sociales afianzan a través de procesos identitarios de ritos y ritualidades, posibilitando su afianzamiento de un sistema a otro, como producto de un legado práctico oral, que en ocasiones se refrenada en fuentes escritas, y que encuentran asidero en la realidad de sus espacios materiales de existencia.

Estas formas espaciales de existir se manifiestan desde el consenso de la simbología, trasladando el simbolismo a un lugar de acción físico con determinadas características y disposición de objetos que sirven para el ejercicio de ese saber. Los agentes le dan una explicación al uso y disposición de estos objetos, que afianza su presencia en el espacio, y desde los cuales se robustece su identidad.

Gracias al arraigo cultural e histórico propio de los saberes de la medicina tradicional, y a pesar de su marginalidad que la medicina occidental o científica les otorga, como empíricos, locales o folclóricos, e invalide sus conocimientos; estos son una alternativa real por la demanda que la población hace de su terapéutica y que alimenta desde sus creencias culturales. Su existencia, por tanto, es el resultado de caminos simbólicos y técnicos, de conceptos de religión y medicina, que por más que se excluyan son alternativas para la búsqueda de la salud, respuesta a la vida, el bienestar, y el equilibrio. Por tanto, la brecha entre la validez científica y saberes populares o tradicionales se entrecruzan por la fuerza de sus existencias en la vida cotidiana de los pueblos.

## Footnotes

1 Este proceso se acompañó del programa de análisis cualitativo ATLAS.ti Versión 9, para ordenar y sistematizar la información obtenida partir de la transcripción de los relatos de vida, por códigos y categorías que facilitaron el posterior el análisis.

2 Según “Un sobandero es un practicante de medicina tradicional cuyos conocimientos han sido adquiridos gracias al trabajo empírico y no a la educación formal, que soluciona problemas ósteo-musculares, como esguinces, desgarres y torceduras, por medio de masajes, acompañados o no de rezos, sobre el área afectada. Los hierbateros y rezanderos son practicantes de la medicina tradicional que también aprenden su oficio gracias a la experiencia y que usan plantas medicinales combinadas con rezos y pases mágicos para solucionar problemas de salud, financieros y emocionales”^(1)^

3 Este apartado se nutre de las observaciones y relatos de los diarios de campo de los investigadores, producto de la observación participante y de sus registros fotográficos en el trabajo de campo.
